# Ectotherm Size‐ and Age‐At‐Maturity in a Warmer, Variable and Resource‐Poor World

**DOI:** 10.1111/ele.70273

**Published:** 2025-12-02

**Authors:** Nathan Frizot, Alexandre Bec, Apostolos‐Manuel Koussoroplis

**Affiliations:** ^1^ CNRS, LMGE Université Clermont Auvergne Clermont‐Ferrand France

**Keywords:** dynamic energy budget, food quality, food quantity, temperature‐size rule, thermal variance

## Abstract

Ectotherms tend to mature at smaller sizes as average temperatures rise, a pattern known as the Temperature‐Size Rule (TSR), which also predicts earlier age at maturity. However, in natural environments, warming is often accompanied by increased thermal variability and limited nutritional resources. Using a bioenergetic model combined with factorial growth experiments on *Daphnia*, we investigated how temperature, food concentration, and food quality (Polyunsaturated fatty acid and sterol content) jointly shape size and age at maturity. We find that poor food quality narrows the upper thermal limit for TSR expression, while low food quantity restricts both upper and lower thermal bounds. Increased thermal variability shifts this range towards cooler temperatures. These findings suggest that the TSR may not hold under ecologically realistic conditions, especially when organisms are close to their thermal optimum where small concomitant increases of resource limitation and temperature variability with warming may lead to smaller yet older individuals at maturity.

## Graphical abstract

The temperature‐size rule (TSR) predicts smaller and earlier maturing ectotherms with warming, but this may not hold under natural conditions. Combining modeling and experiments on Daphnia, we show that food quality, quantity and thermal variability jointly constrain TSR expression, potentially leading to smaller yet older individuals at maturity.

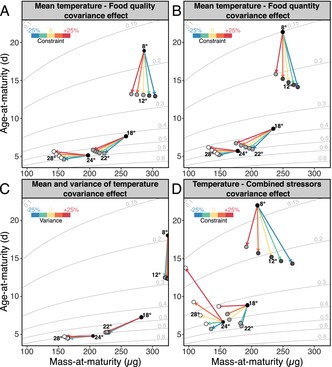

## 1 Introduction

**FIGURE 1:**
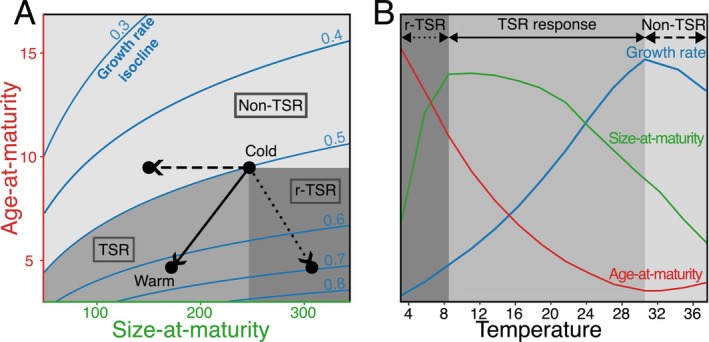
Theoretical ectotherm life history (size‐ and age‐at‐maturity) and growth rate responses to temperature increase. (A) Life‐history response patterns in the age‐size phase space. Isoclines indicate levels of somatic growth rate, calculated from combinations of size‐ and age‐at‐maturity (see Equation 12 in text). The arrows are hypothetical responses to an increase in temperature. The temperature‐size rule (TSR, medium grey area) predicts a combination of decreased size‐ and age‐at‐maturity and increased growth rate at warmer temperatures. Other potential response types include reverse‐TSR (r‐TSR; dark grey area) where age decreases while size increases or non‐TSR responses (light grey area) where growth rate decreases. (B) Thermal range of life‐history response types. Coloured lines illustrate the hypothetical thermal reaction norms for size‐, age‐ and growth rate‐at‐maturity for an exponentially growing juvenile ectotherm. Here, the temperature limits of the different response types are defined by the local slope of the reaction norms. Note that in practice, experiments might sample temperatures that overlap different regions. Here, we hypothesize that because the shape of thermal reaction norms depends on other environmental factors, covariance between those factors and temperature can alter the life‐history response type within a given temperature range.

Ectotherms often tend to become smaller as mean temperatures rise. This pervasive pattern of body size‐at‐maturity and asymptotic size is considered the third universal response of ectotherms to global warming along with phenological changes and geographical distribution shifts (Daufresne et al. 2009). The temperature‐size rule (TSR) is a special case of size‐ and age‐at‐ontogenetic stage (e.g., maturity) thermal reaction norms. Angilletta et al. (2004) define TSR as the situation that satisfies three conditions: smaller size‐at‐maturity, lower age‐at‐maturity and higher somatic growth rate at warmer temperatures (Figure 1). However, size‐at‐maturity does not always decrease with temperature. Another documented pattern is the reverse‐TSR (r‐TSR), which occurs when size‐at‐maturity and growth rate increase with temperature, while age‐at‐maturity decreases (Figure 1) (Kingsolver et al. 2007). All other possible patterns involving a decrease of growth rate with temperature (Figure 1) are referred to here as non‐TSR.

One of the most prominent adaptive explanations centers on a size‐time trade‐off, where warm conditions favour faster reproduction (lower age‐at‐maturity) over larger size‐at‐maturity to mitigate higher mortality risks (Audzijonyte et al. 2022; Bruijning et al. 2018; Verberk et al. 2021). From an individual energy budget perspective, the mechanistic basis of the TSR is a greater allocation of energy to development than to growth as temperature rises. By definition, the ability of organisms to follow the TSR is limited to the thermal range where growth rate increases with temperature (Walczyńska et al. 2016). This range should therefore depend on the environmental context. For example, reduced resource availability has been shown to decrease the optimal temperature and upper thermal limits for growth, altering the range in which the TSR is observed (Huey and Kingsolver 2019; Verberk et al. 2021). Similarly, resource quality also modulates the intensity of the TSR (i.e., how much size changes per temperature degree) and even reverses it (i.e., reverse‐TSR) (Diamond and Kingsolver 2010; Lee et al. 2015; Torres and Giménez 2020). Beyond resources, temperature variance should also affect the temperature range over which growth rate increases with temperature. Jensen's inequality predicts that the net energy gain of an ectotherm in thermally varying environments should differ from that in constant environments with the same mean temperature as long as net energy gain is a nonlinear function of temperature (Denny 2019; Huey and Kingsolver 2019; Jensen 1906; Koussoroplis et al. 2017). Hence, by acting on the energy budget, thermal variance could act on size‐at‐age patterns. However, this hypothesis remains to be tested.

Global warming is expected to induce simultaneous changes in mean temperature but also in temperature variability (Easterling et al. 2000, 1997; Kotz et al. 2021; Wang and Dillon 2014), as well as in resource availability (Boyce et al. 2010; Kraemer et al. 2017; Zhao and Running 2010) and quality (Birnie‐Gauvin et al. 2017; Hallam and Harris 2023; Hardison and Eliason 2024). In a warming world, rising temperatures can act directly on ectotherm nutrition by inducing changes in primary production. In oceans net primary production is expected to decline, particularly at lower latitudes (Ryan‐Keogh et al. 2025; Zhang et al. 2025). On land, global net primary production is expected to increase, although locally, drought and nitrogen limitation could reverse the trend (Zhang et al. 2025; Zhao and Running 2010). In lakes, increased temperatures lead to increased stratification of the water column (Woolway et al. 2021), creating nutrient limitation that decreases phytoplankton productivity (O'Reilly et al. 2003). These warmer temperature and nutrient decline conditions change the dominant species of phytoplankton towards species of lower food quality such as cyanobacteria (Kosten et al. 2012; Reinl et al. 2021). These species lack polyunsaturated fatty acids (PUFAs) and phytosterols (Bec et al. 2006; Martin‐Creuzburg et al. 2006) which are essential biomolecules for many metazoans (Twining et al. 2021). In oceans too, increasing surface temperatures strongly correlate with decreasing proportions of long‐chain omega‐3 PUFAs in phytoplankton (Hixson and Arts 2016). On land, the increase in CO_2_ and temperature typically causes a phenomenon known as *nutrient dilution* involving increased plant biomass with lower nitrogen, phosphorus and protein concentrations thereby limiting herbivore growth (Kaspari and Welti 2024). Finally, temperature variance is expected to change along with increasing mean temperatures (Bernhardt et al. 2020; Dillon et al. 2016; Vasseur et al. 2014). In regions where temperature variance is expected to rise with mean temperature, the increase in daily temperature variability could reach up to 100% by the end of the century (Kotz et al. 2021).

These lines of evidence suggest that global change is driving not only towards a warmer world but also one that is more variable and resource‐depleted, especially in aquatic environments, emphasizing the importance of examining temperature effects on size‐at‐age responses not just across different environmental contexts but also in relation to shifting environmental conditions. This urges the need to account for the covariation between temperature gradients and environmental context to predict the diversity of size‐ and age‐at‐maturity responses to temperature observed in nature (Daufresne et al. 2009; Morita and Fukuwaka 2007). Indeed, understanding when and why these different patterns hold is crucial, as shifts in size‐ and age‐at‐maturity affect reproductive output, mortality risk and trophic interactions, ultimately influencing population dynamics and ecosystem structure (Sentis et al. 2017; Woodward et al. 2005). In that aim, it is necessary to develop theoretical frameworks (Audzijonyte et al. 2022; Hoefnagel et al. 2018; Richard et al. 2024) that explain the size‐at‐age response continuum within and across environmental contexts. Here, we relax some assumptions of the standard Dynamic Energy Budget (DEB) theory (Kooijman 2009) on the thermal dependence of energy uptake, metabolism and allocation. This enables us to capture TSR patterns and to predict the continuum of ectotherm size‐ and age‐at‐maturity responses within and across nutritional and thermal variance contexts. We tested some predictions using *Daphnia magna* , a freshwater filtering crustacean, as a model species (Ebert 2022) in a series of factorial growth experiments in which food concentration, quality (PUFAs and sterol availability) as well as thermal mean and variance were manipulated. The aim of the study is to see how the covariance between temperature and other environmental factors may underlie changes in size‐age patterns in ectotherms. Our study provides a mechanistic explanation of the diverse patterns of size‐ and age‐at‐maturity observed under various environmental contexts. Furthermore, it offers predictions for more ecologically relevant scenarios where resource quantity and quality as well as thermal variance change along with mean temperature.

## 2 Materials and Methods

### 2.1 Model Overview

We used Dynamic Energy Budget (DEB) theory (Kooijman 2009) to predict the thermal reaction norms of age‐ and size‐at‐maturity of an ectotherm within and across environmental contexts. Our DEB model (Extended Figure S1) accounts for energy acquisition through feeding and allocation between different energy demanding processes to simulate growth, development and reproduction over an individual's life‐time. Energy flows within the organism depend on three state variables: structural volume (V), energy reserve (E) and maturity status/reproductive buffer (R).

At the reference temperature $T^{*}=20^{{}^{\circ}}C$, energy reserves are taken‐up from available resource at a rate ${\dot{p}}_{A}$ such that:

(1) $${\dot{p}}_{A}=\left\{{\dot{p}}_{\mathrm{AM}}\right\}V^{\frac{2}{3}}f\left(X\right)$$

where $\left\{{\dot{p}}_{\mathrm{AM}}\right\}$ is the maximum surface‐specific uptake rate (J.cm^−2^d^−1^) and $f\left(X\right)=\frac{X}{X+k_{s}}$ the scaled functional response to the resource concentration X (mg of algal C.L^−1^) with a half saturation coefficient $k_{s}$.

The energy is mobilised from the reserve at a rate ${\dot{p}}_{C}$ following:

(2) $${\dot{p}}_{C}=E\left(\dot{v}\left[E_{G}\right]V^{\frac{2}{3}}+{\dot{p}}_{S}\right)/\left(\left[E_{G}\right]V+\mathrm{\kappa E}\right)$$

where $\dot{v}$ is the energy conductance (cm.d^−1^), $\left[E_{G}\right]$ the specific cost of structure (J.cm^−3^), and ${\dot{p}}_{S}$ is the structural maintenance cost such that:

(3) $${\dot{p}}_{S}=\left[{\dot{p}}_{M}\right]V$$

where $\left[{\dot{p}}_{M}\right]$ is the volume‐specific somatic maintenance rate at $T^{*}$ (J.cm^−3^.d^−1^). In Equation 2, κ is the fraction of mobilised energy allocated to somatic maintenance and growth. The remaining fraction 1−κ is allocated to maturity maintenance and maturation (before puberty) or reproduction (after puberty). As in many theoretical models (Day and Rowe 2002; Nilsson‐Örtman and Rowe 2021), maturation in DEB theory is also threshold‐driven. Maturation is viewed as a life‐history transition that occurs once an organism has accumulated sufficient energy to ‘pay’ developmental cost. Maturation is not timed by chronological age but is linked to the energetic state of the organism. When sufficient energy reserves have been allocated to development the organism transits to the next phase. Hence, in the model the transition from juvenile to adult occurs when the cumulative energy allocation to maturity reaches some specific fixed threshold $R_{p}$. Unlike classic DEB models, we added a minimal structural mass threshold for maturation $V_{p}$ in agreement with Nilsson‐Örtman and Rowe (2021) in order to avoid unrealistically small maturation sizes at temperature extremes. When both thresholds are reached, the 1−κ fraction of mobilised reserve energy serves to build the reproductive buffer. The energy fluxes in Equations (1), (2), (3) determine the dynamics of the three state variables as follows:

Energy reserve:

(4) $$\frac{\mathrm{dE}}{\mathrm{dt}}={\dot{p}}_{A}-{\dot{p}}_{C}$$

Somatic growth:

(5) $$\frac{\mathrm{dV}}{\mathrm{dt}}=\left(\kappa {\dot{p}}_{C}-{\dot{p}}_{s}\right)/\left[E_{G}\right]$$

Maturation/Reproductive buffer:

(6) $$\frac{\mathrm{dR}}{\mathrm{dt}}=\left(1-\kappa \right){\dot{p}}_{C}-{\dot{k}}_{j}R$$

where ${\dot{k}}_{j}$ is the reproductive maintenance coefficient (d^−1^) at $T^{*}$.

### 2.2 Temperature Dependence of Physiological Rates and Energy Allocation

In the standard DEB model, physiological rates (denoted by a dot above the character) scale in the same way with temperature. Yet, theoretical studies show that to capture TSR patterns, some assumptions of the standard DEB model have to be relaxed (Hoefnagel et al. 2018; Kearney and White 2012; Richard et al. 2024). First, energy uptake and metabolism (i.e., energy mobilisation and maintenance rates) need to have different thermal dependencies. Here, we followed Kearney and White (2012) assuming that uptake rate has a unimodal relation to temperature (Sharpe‐Schoofield model) whereas maintenance rates increase monotonically (Arrhenius model). Hence, in our DEB model $\left\{{\dot{p}}_{\mathrm{AM}}\right\}$ is multiplied by:

(7) $$q_{A}=\frac{e^{E_{a}\left(\frac{1\;}{kT^{*}}-\frac{1}{\mathrm{kT}}\right)}}{1+e^{E_{d}\left(\frac{1}{kT_{d}}-\frac{1}{\mathrm{kT}}\right)}}$$

where k the Boltzman constant (8.617 × 10^−5^ eV.K^−1^), $E_{a}$ and $E_{d}$ the activation and deactivation energies (0.65 and 1.89 eV, respectively) and $T_{d}$ the temperature at which 50% deactivation is reached (302.15 *K* = 29°C). All other physiological rates ($\dot{v},\left[{\dot{p}}_{M}\right],{\dot{k}}_{j})$ are multiplied by:

(8) $$q_{M}=e^{E_{a}\left(\frac{1}{kT^{*}}-\frac{1}{\mathrm{kT}}\right)}$$

There is no consensus on how the differential temperature dependence of uptake and maintenance should be modelled (Hoefnagel et al. 2018; Richard et al. 2024). Our approach has the advantage to be in agreement with experimental observations of ingestion and respiration rates in daphnids (Kibby 1971; Wiggins and Frappell 2000) and other ectotherms (Lemoine and Burkepile 2012).

Kearney and White (2012) suggested that the DEB assumption of a constant allocation rule κ across temperatures should also be relaxed. In agreement with this suggestion, we allow κ to vary between two boundaries $\left(\kappa_{\min},\kappa_{\max}\right)$ following a sigmoid relationship:

(9) $$\kappa \left(T\right)=\kappa_{\max}-\frac{\kappa_{\max}-\kappa_{\min}}{1+e^{-\lambda \left(T-\hat{T}\right)}}$$

where $\hat{T}$ is the inflexion temperature and λ the parameter controlling the stiffness of the transition. Plastic responses of κ to temperature are not obligatory to yield TSR patterns in energy budget models when uptake and maintenance have different sensitivities (Hoefnagel et al. 2018; Richard et al. 2024). However, Hoefnagel et al. (2018) obtained better model fits to their data when allowing for temperature dependence of κ. Further lines of evidence in favour of plasticity in the parameter κ is that *Daphnia* are able to adjust their age‐ and size‐at‐reproduction schedules to the identity of their predators (Riessen 1999) probably as an adaptive response to their prey selection strategies.

### 2.3 Model Parametrization and Implementation

Our objective here is not to estimate model parameters but to explore how correlated environmental changes affect size‐ and age‐at‐maturity patterns in a model ectotherm. Nevertheless, we tried to parametrize the model as close as possible to *Daphnia magna* using mostly published parameter values for this species (Table 1). Food quality effects were implemented by altering maintenance costs $\left[{\dot{p}}_{M}\right]$ and ${\dot{k}}_{j}$. The minimal and maximal values used for $\left[{\dot{p}}_{M}\right]$ were derived from Ruiz et al. (2021) and correspond to the standard metabolic rates observed for individuals fed high‐quality *Cryptomonas* algae and low‐quality *Synechococcus* cyanobacteria, respectively. Note that the range of values we used (330–740 J.cm^−3^.d^−1^) is below the value estimated in published *D. magna* DEB models (1200 J.cm^−3^.d^−1^; Kooijman and Gergs 2023). Similarly to $\left[{\dot{p}}_{M}\right]$, ${\dot{k}}_{j}$ was also allowed to vary by 100% between the highest food quality (0.05 d^−1^) and the lowest one (0.1 d^−1^). For this parameter too, the range of values used is below the value estimated in published *D. magna* DEB models (0.25 d^−1^; Kooijman and Gergs 2023) which yielded unreasonably old ages at first reproduction (not shown). In Equation 9 the range covered by $\kappa_{\min}$ (0.6) and $\kappa_{\max}$ (0.7) is very close to the published 0.58 cm.d^−1^ (Kooijman and Gergs 2023) whereas $\hat{T}$ and λ were arbitrarily chosen to match the data (sensitivity analysis shown in Extended Figure S2).

**TABLE 1:** Model's parameters and equations. The model has been parameterized for *Daphnia magna* .

Symbol	Description	Differential equation	Unit
State variables			
E	Reserve	$\frac{\mathrm{dE}}{\mathrm{dt}}={\dot{p}}_{A}-{\dot{p}}_{C}$	J
V	Soma	$\frac{\mathrm{dV}}{\mathrm{dt}}=\left(\kappa {\dot{p}}_{C}-{\dot{p}}_{s}\right)/\left[E_{G}\right]$	cm^3^
R	Development $\left(R<R_{p}\right)$, Reproduction buffer $\left(R>R_{p}\right)$	$\frac{\mathrm{dR}}{\mathrm{dt}}=\left(1-\kappa \right){\dot{p}}_{C}-{\dot{k}}_{j}R$	J

Fluxes		
Name	Equation	Unit
Energy uptake from food	${\dot{p}}_{A}=\left\{{\dot{p}}_{\mathrm{AM}}\right\}V^{2/3}f\left(X\right)$	J.d^−1^
Mobilisation from reserve	${\dot{p}}_{C}=E\left(\dot{v}\left[E_{G}\right]V^{\frac{2}{3}}+{\dot{p}}_{S}\right)/\left(\left[E_{G}\right]V+\mathrm{\kappa E}\right)$	J.d^−1^
Structural maintenance costs	${\dot{p}}_{S}=\left[{\dot{p}}_{M}\right]V$	J.d^−1^

Functions	
Description	Equation
Functional response	$f\left(X\right)=\frac{X}{X+X_{s}}$
Temperature scaling factor for $\left\{{\dot{p}}_{\mathrm{AM}}\right\}$	$q_{A}=\frac{e^{E_{a}\left(\frac{1\;}{kT^{*}}-\frac{1}{\mathrm{kT}}\right)}}{1+e^{E_{d}\left(\frac{1}{kT_{d}}-\frac{1}{\mathrm{kT}}\right)}}$
Temperature scaling factor for $\dot{\dot{v},\left[{\dot{p}}_{M}\right],{\dot{k}}_{j}}$	$q_{M}=e^{E_{a}\left(\frac{1}{kT^{*}}-\frac{1}{\mathrm{kT}}\right)}$
Temperature scaling for κ	$\kappa \left(T\right)=\kappa_{\max}-\frac{\kappa_{\max}-\kappa_{\min}}{1+e^{-\lambda \left(T-\hat{T}\right)}}$

Symbol	Description	Value at $T_{r}$	Unit	Source
*Temperature‐dependent parameters*				
$\left\{{\dot{p}}_{\mathrm{AM}}\right\}$	Max Surface‐specific uptake rate	313	J.cm^−2^.d^−1^	Kooijman and Gergs (2023)
κ	Allocation of assimilated energy to soma	0.6 (min) 0.7 (max)	/	Kooijman and Gergs (2023)
*Temperature‐ and food quality‐dependent parameters*				
$\left[{\dot{p}}_{M}\right]$	Volume‐specific somatic maintenance rate at $T^{*}$	330 (high quality) 740 (low quality)	J.cm^−3^.d^−1^	Ruiz et al. (2021)
${\dot{k}}_{j}$	Reproductive maintenance coefficient at $T^{*}$	0.05 (high quality) 0.1 (low quality)	d^−1^	Kooijman and Gergs (2023)
*Fixed parameters*				
$\left[E_{G}\right]$	Specific cost for soma	4400	J.cm^−3^	Kooijman and Gergs (2023)
$E_{a}$	Activation energy	0.65	eV	Lindmark et al. (2022)
$E_{d}$	Deactivation energy	1.89	eV	Lindmark et al. (2022)
$R_{p}$	Cumulative energy invested to reach puberty	1.03	J	Kooijman and Gergs (2023)
$V_{p}$	Minimum structural mass to reach puberty	90	μg	Personal observation
$k_{s}$	Half‐saturation coefficient	0.5	mgC.L^−1^	Arbitrary
$\hat{T}$	Inflexion temperature	20	°C	Arbitrary
λ	Stiffness parameter	0.6	/	Arbitrary
$T^{*}$	Reference temperature	20	°C	Arbitrary
$T_{d}$	Temperature of 50% deactivation	29	°C	Arbitrary
$\dot{v}$	Energy conductance at $T^{*}$	0.18	cm.d^−1^	(Kooijman and Gergs 2023)

In daphnids, determining age‐at‐maturity requires frequent microscopic observation of the ovaries which is logistically unfeasible for experiments involving hundreds of individuals. Usually, maturity is diagnosed when the eggs are laid in the brood pouch. To account for this, we modelled the duration of the first adult instar δ, that is, the duration between the moment $R_{p}$ is reached (maturity in the model) and the moment the eggs are laid (experimentally observed maturity) as a function of temperature:

(10) $$\delta \left(T\right)=\frac{\delta_{T^{*}}\;}{e^{E_{a}\left(\frac{1}{kT^{*}}-\frac{1}{\mathrm{kT}}\right)}}$$

assuming that $\delta_{T^{*}}=2\;\text{days}$ (i.e., duration at reference temperature; personal observation). Hence, the age of *observed* maturity is delayed by $\delta \left(T\right)$ relative to *real* age‐at‐maturity. Once the model predicts clutch arrival, the mass‐at‐maturity is defined as the sum of structure, reserve and clutch after using the appropriate conversion coefficients (Table 1): structural volume was converted to mass by assuming a mass density $d_{m}=19\times 10^{4}$ μg.cm^−3^ (Kooijman and Gergs 2023). Reserve was converted by assuming energy density of $d_{e}=25\times 10^{-3}$ J.μg^−1^ (Schindler 1968).

To match experimental treatments (see below), temperature fluctuations were simulated as 24 h‐period square waves using:

(11) $$T\left(t\right)=\bar{T}+A\times \mathrm{sgn}\left(\sin\left(2\mathrm{\pi t}\right)\right)$$

where A is the temperature amplitude in °C. The differential equations of the model were integrated using the *ode* solver in the *deSolve* R package (Soetaert et al. 2010).

### 2.4 Simulations

To map response patterns (TSR, reverse‐TSR, and non‐TSR) across environmental contexts, we simulated size‐ and age‐at‐maturity for a factorial grid of mean temperature and factors of interest (food quantity, quality and temperature diurnal amplitude). Juvenile growth rate was then calculated from the simulation results (Equation 12). Local slopes along the temperature axis were extracted from the resulting response surfaces and were used to classify response patterns in the temperature‐factor of interest space (see Extended Methods: Supporting Information).

We also simulated scenarios of covariation between temperature gradients and environmental context across three temperature ranges (8°C–12°C, 18°C–22°C and 24°C–28°C) representing the lower, middle, and upper portions of *Daphnia*'s thermal performance curve, respectively. In all simulations, food quantity (X) was fixed at 1 mgC.L^−1^, food quality parameters ($\left[{\dot{p}}_{M}\right]$ and ${\dot{k}}_{j}$) were set to the median values from Table 1, and diurnal temperature amplitude was held constant at 2.5°C as reference. To test the effects of environmental covariation, we simulated scenarios where the values of single factors changed from baseline values at rates ranging from −6.25% to +6.25% per °C. This corresponds to a total change of ±25% over an arbitrarily chosen 4°C increase. While many combinations are possible when multiple factors covary, we focused (for reasons outlined in the Introduction) on a biologically motivated scenario in which food quantity covaries positively with food quality and negatively with diurnal temperature amplitude. Simulation outcomes were visualised in age–size phase space (as in Figure 1A), and response types were evaluated visually.

### 2.5 Organisms' Maintenance and Food Preparation

We used a clone of *Daphnia magna* isolated from a pond in the Auvergne region (France). Organisms were reared in Volvic water at 20°C under a natural day/night cycle and fed a saturating diet consisting of an 80:20 mix of *Chlamydomonas reinhardtii* and *Cryptomonas* sp. (3 mgC.L^−1^) over multiple generations. Phytoplankton species were grown on WC medium (Guillard 1975) at 20°C in batch cultures in 5 L aerated vessels under permanent light with a dilution rate of 0.25 d^−1^. Food was prepared by centrifugation and dilution in Volvic water. The carbon concentration was estimated by spectrophotometry (800 nm) using a pre‐established carbon concentration regression.

### 2.6 Experimental Set‐Up

We conducted a full‐factorial experiment crossing two levels of mean temperature (20°C, 28°C), thermal variance (constant vs. fluctuating with 10°C amplitude, 24 h period), food concentration (3 vs. 0.4 mgC.L^−1^), and food quality (*Cryptomonas* sp. vs. *Synechococcus* sp. mix), resulting in 16 treatment combinations. Monoclonal *Daphnia magna* neonates (10 h‐old, third clutch) were pre‐acclimated to their respective mean temperature and randomly assigned to treatments (four replicate jars per treatment). Each jar held 8 individuals in 600 mL (high food) or 150 mL (low food) of Volvic water, renewed daily with food at *ad libitum* levels (≥ 0.7 mgC.L^−1^) (Müller‐Navarra and Lampert 1996). In low food treatments, a flow‐through system (60 mL.h^−1^) maintained constant food concentration.

Diet quality was manipulated using *Cryptomonas* sp. (high in PUFAs and sterols) or a 90:10 mix of *Synechococcus* sp. and *Chlamydomonas reinhardtii* (low in PUFAs/sterols). Constant‐temperature treatments were maintained at 20°C or 28°C, while fluctuating treatments cycled ±5°C around the mean every 12 h. Individuals were raised until maturity (4–21 days depending on treatment), then dried (48 h) and weighed individually. Body mass was used as a proxy for size. Juvenile specific growth rate *g* (d^−1^) was calculated as:

(12) $$g=\frac{\ln\left(\frac{m_{M}}{m_{O}}\right)}{a_{M}}$$

where $m_{O}$ and $m_{M}$ are neonate mass and mass‐at‐maturity and $a_{M}$ is age‐at‐maturity.

### 2.7 Data Analysis

To assess the combined effects of temperature, thermal variance, food quality, and food quantity on mass‐at‐maturity, age‐at‐maturity, and somatic growth rate, we applied mixed‐effects models using the *lme4* package (Bates et al. 2015). ANOVA and pairwise comparisons of estimated marginal means were conducted for selected conditions.

Model performance was evaluated by comparing experimental and predicted values using Root Mean Squared Error (RMSE) and Mean Bias Error (MBE), the latter quantifying systematic over‐ or underestimation. To facilitate comparison across traits, RMSE and MBE were normalised by dividing each metric by the mean observed value of the corresponding trait. All analyses were performed in R v4.1.2 (R Core Team 2025) with an alpha level of 0.05.

## 3 Results

### 3.1 Modelling Mass‐, Age‐At‐Maturity and Growth Rate Within Different Environments

**FIGURE 2:**
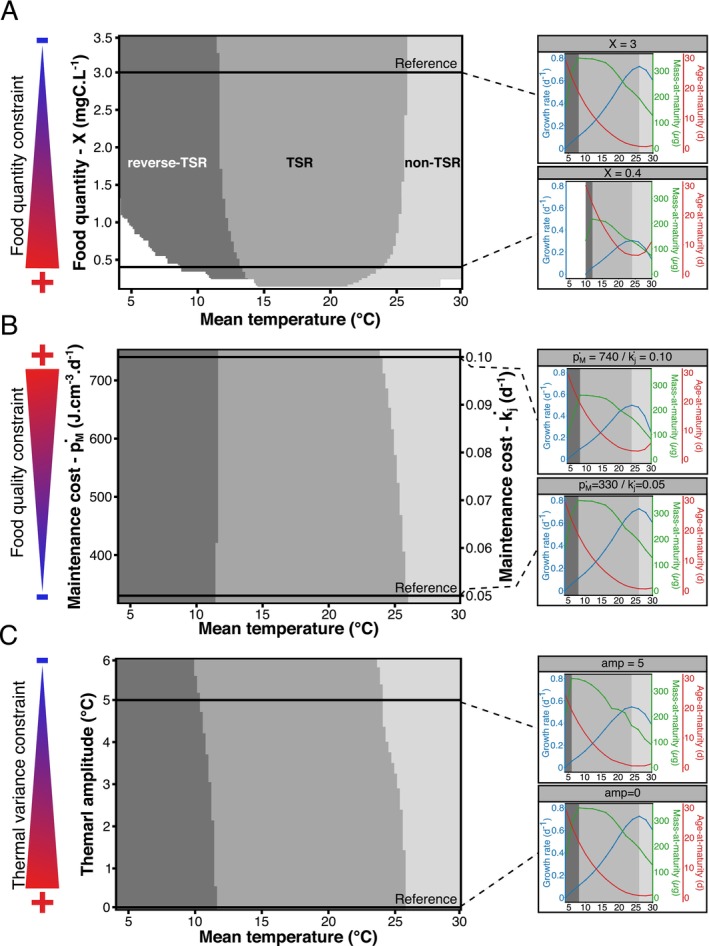
Modelled thermal range of life‐history response patterns for gradients of (A) food quantity, (B) maintenance costs (modulated by food quality constrains) and (C) thermal variance. Side panels illustrate thermal reaction norms of life history traits for specific constraint levels: Low food quantity environment (X = 0.4), low food quality environment (i.e., high maintenance costs: ${\dot{p}}_{M}=740$ and ${\dot{k}}_{j}$ = 0.1) and diurnally fluctuating environment (Amp = 5). The white area illustrates when the model did not succeed to predict maturity in the given time frame. The reference panel represents a non‐stressful environment, with a high food concentration (X = 3), high food quality (i.e., low maintenance costs: ${\dot{p}}_{M}=330$ and ${\dot{k}}_{j}$ = 0.05) and constant temperature (Amp = 0).

The model predicts that TSR should be restricted to a specific range of intermediate temperatures. Below this range (colder temperatures), the model predicts a reverse‐TSR pattern as the slope of the thermal reaction norm of mass‐at‐maturity becomes positive (Figure 2A‐reference side panel). At hotter temperatures, it predicts a non‐TSR pattern explained by an inversion of the slope of the age‐at‐maturity reaction norm to temperature. Consequently, above the inversion temperature, the growth rate begins to decrease with temperature. When food quantity becomes constraining, the model predicts a general shift towards smaller size and older age‐at‐maturity (Figure 2A‐main panel). The slope inversion point of mass‐at‐maturity shifts towards higher temperatures whereas the point where age‐at‐maturity begins to increase with temperature is shifted towards colder temperatures. As a result, the thermal range of TSR narrows from both cold and warm fronts.

When nutritional quality is constraining, the slope inversion point of mass‐at‐maturity remains almost unchanged while the point where age‐at‐maturity begins to increase with temperature is shifted towards colder temperatures (Figure 2B‐side panel). Thus, the thermal range of TSR only narrows from its warm front (Figure 2B‐main panel).

In a thermally variable environment, the model predicts that the slope inversion points of mass‐ and age‐at‐maturity both shift towards colder temperatures (Figure 2C‐side panel). As a consequence, the width of the TSR thermal range remains almost unaffected but shifts towards colder temperatures (Figure 2C‐main panel).

### 3.2 Agreement Between Model and Observation Data

**FIGURE 3:**
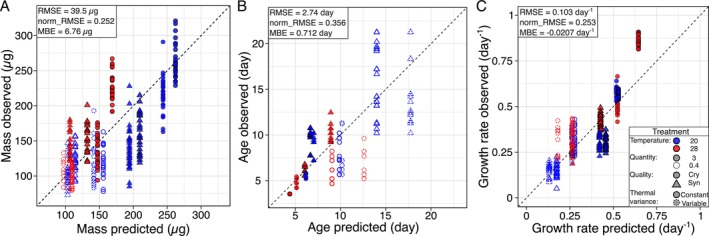
Observed results versus predicted results for (A) mass, (B) age and (C) growth rate. Observed data is represented on the y‐axis and predicted data is represented on the *x*‐axis. The black dashed line represents the 1:1 ratio; points closer to this line indicate a stronger agreement between experimental data and model predictions. The treatment section indicates the combination of the four factors tested: mean temperature, food quality (Cry = *Cryptomonas* sp., high food quality; Syn = *Synechococcus* sp., low food quality), thermal variance (amplitude: ±5°C around the mean) and food quantity. RMSE (root mean square error) quantifies the overall prediction error magnitude, expressed in the same units as the response variable. norm‐RMSE is RMSE normalised by the mean of observed values, ranging from 0 (perfect prediction) to 1 (high error relative to the mean). MBE (mean bias error) is the mean error between predicted and observed values; positive value indicates overestimation of predictions and negative value indicates underestimation of predictions.

In our 2 temperatures × 2 food qualities × 2 food concentrations × 2 variance regimes factorial experiment we found strong interactive effects of the manipulated factors on size‐ and age‐at maturity as well as growth rate of *Daphnia magna* (Extended Figure S3, Extended Tables S1–S4). In the treatments combining high mean temperature, low food quantity and quality treatments, daphnids died before maturity. No mortality was observed under any of the other conditions. For remaining treatments, the model predictions agree with experimental results (Figure 3A–C). The prediction accuracy is comparable across factorial combinations with few exceptions with biases as high as −58.3 μg (−25.7%) for mass, 5.91d (+88.4%) for age and −0.217d^−1^ (−25.2%) for growth rate (Extended Figure S4).

### 3.3 Predicting Size‐ and Age‐At‐Maturity Trajectories in Scenarios of Environmental Covariance

**FIGURE 4:**
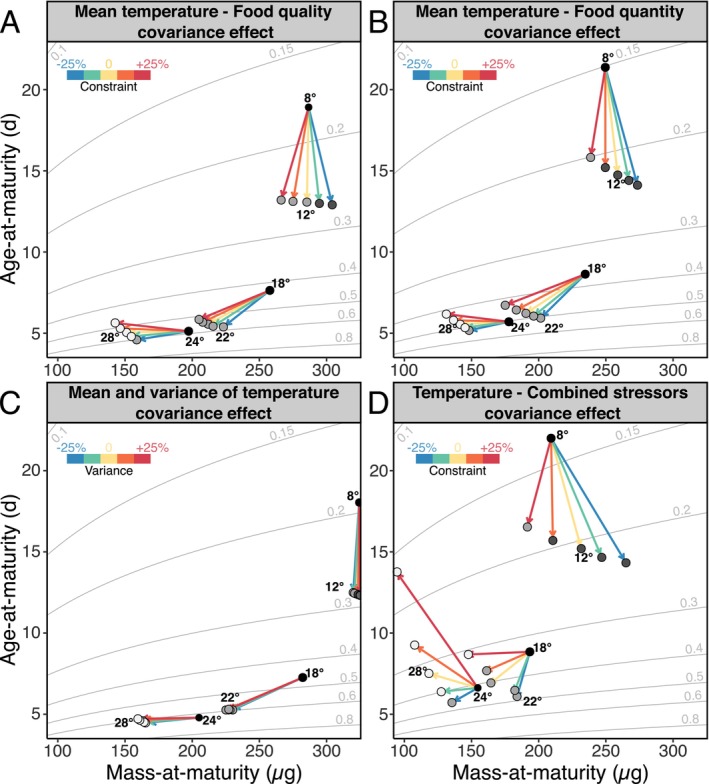
Modelled effects of covariation between temperature and other environmental factors on mass‐ and age‐at‐maturity. Arrows indicate life history responses to 4°C increases in temperature under different covariation scenarios. The yellow arrows indicate scenarios without covariation. The other colours indicate scenarios where the constraint from (A) food quality (controlled by $\left[{\dot{p}}_{M}\right]$ and ${\dot{k}}_{j}$), (B) food quantity (controlled by X), (C) temperature variance (controlled by diurnal amplitude) and (D) a combination of all three factors constraints either increases (orange, red) or decreases (green, blue) with temperature. Covariation rates are 3.125%.°C^−1^ (orange, green) or 6.25%.°C^−1^ (red, blue), resulting to a total change of 12.5% and 25% respectively. Point colours indicate the different response patterns: Reverse‐TSR (dark grey), TSR (medium grey) or non‐TSR (light grey). The isoclines illustrate the somatic ponderal growth rate calculated from size‐ and age‐at‐maturity assuming exponential growth (see Equation 12 in text).

The model predicts strong sensitivity to covariance between temperature and resource constraints (Figure 4A,B). At lower temperatures (8°C–12°C), covariance alters the slope of the relationship between temperature and size‐at‐maturity. Modest changes in the direction and strength of maintenance costs (i.e., implying changes in food quality) or food quantity variation with temperature (from −3.125% to +3.125% per °C) can shift the response from TSR to reverse‐TSR (r‐TSR).

In the intermediate temperature range (18°C–22°C), a TSR pattern is maintained across all scenarios, but the slopes relating size and age at maturity to temperature differ depending on the nature of the covariance. At the highest temperatures, TSR patterns are observed only in cases where the covariance between food and temperature most strongly reduces constraints. In all other scenarios, individuals mature at smaller sizes and older ages as temperature rises, indicating a breakdown of the TSR.

The effects of temperature variance (Figure 4C) are weaker. The tested changes in amplitude (2.5°C ± 6.25% per °C) have limited influence, mainly affecting responses at temperature extremes. At low temperatures, negative covariance between mean temperature and variance reduces size and age at maturity, while positive covariance produces the opposite effect at high temperatures.

When all environmental stressors covary simultaneously (Figure 4D), their effects are amplified. Even within the intermediate thermal range, the TSR pattern disappears when food quantity or quality decreases and variance increases at rates of 6.25% per °C. In these conditions, individuals mature later and at smaller sizes.

## 4 Discussion

The temperature‐size rule, is one of the most widespread responses to temperature observed in nature (Daufresne et al. 2009; Gardner et al. 2011). Identifying the environmental conditions in which this pattern is altered is key to understanding the generality of the rule and its repercussions on the dynamics and structure of ecosystems. Our model captures all size and age responses at maturity to increased temperature that have been observed in natural and experimental contexts (Figures 2 and 4), including TSR, situations where only age or size are affected as well as reverse‐TSR (Bigler et al. 1996; Diamond and Kingsolver 2010; Sainte‐Marie et al. 2021; Torres and Giménez 2020). Most importantly, the model predicts how environmental conditions modulate size‐at‐age patterns, via the organism's energy budget.

### 4.1 Integrating Nutritional Constraints to the TSR

An important issue in exploring the TSR is its robustness to variations in the nutritional environment (Berrigan and Charnov 1994; Diamond and Kingsolver 2010; Lee et al. 2015). Resource may vary in terms of quantity and/or quality and both aspects interact with temperature to affect ectotherm life‐history traits (Diamond and Kingsolver 2010; Giebelhausen and Lampert 2001; Masclaux et al. 2009; Torres and Giménez 2020). Yet, our understanding of the TSR from a nutritional perspective is limited to empirical observations and hindered by the overwhelmingly multidimensional nature of resource quality (Danger et al. 2022; Diamond and Kingsolver 2010; Jonsson et al. 2013; McFeeters and Frost 2011). Our model offers a simple and tractable integration of dietary constraints into the organism's energy budget: quantitative constraints act on the resource uptake rate whereas qualitative ones act on maintenance rates. The simulations show that nutritional constraints acting on maintenance costs (i.e., food quality) tend to reduce the thermal range where TSR emerges by decreasing its upper limit. On the other hand, nutritional constraints on energy uptake (food quantity) reduce the range by acting on both upper and lower limits. This also implies that the thermal range where reverse‐TSR emerges is shifted towards higher temperatures. This finding could explain the observations on *Manduca sexta* caterpillars for which the TSR pattern is reversed when submitted to nutritional constraints (Diamond and Kingsolver 2010). The authors considered the nutritional constraint as ‘qualitative’ without explaining the exact nature of the constraint. In our model however, the reversal of TSR within the same temperature range would rather correspond to a quantitative constraint as it is produced by a reduction in energy uptake.

Other observational studies show that nutrition modulates the TSR, in particular the slope of mass‐at‐age with temperature (Lee et al. 2015; Torres and Giménez 2020). Torres and Giménez (2020) observed that in *Carcinus maenas* larvae the typical temperature‐induced reduction in size and age at metamorphosis occurred only under food restriction. In contrast, *ad libitum*‐fed larvae showed a decrease in age at metamorphosis with increasing temperature, but size remained unchanged. Our model mirrors these empirical findings. It predicts that within the thermal range associated with the TSR, the relationship between size and temperature can shift—from nearly flat (but slightly negative) to strongly negative—as temperature increases. This suggests that when experimental temperatures fall within the ‘flat’ segment of this range, size reductions may go undetected, even though developmental timing is affected. The sensitivity analysis (Extended Figure S2) highlights that the breadth of this flat region is controlled by the parameter *λ*, which determines the steepness of the decline in the proportion of energy allocated to growth (*κ*) with temperature. Higher values of *λ* produce steeper declines, resulting in a broader temperature range over which size at maturity remains stable—as in the *ad libitum*‐fed larvae observed by Torres and Giménez (2020), where only age at metamorphosis declined. When simulating food restriction, the flat region narrows (Figure 2), and within the same temperature range, the slope of size with temperature becomes distinctly more negative. This transition matches the pattern seen under food‐limited conditions in the Torres and Giménez (2020) experiments, reinforcing the model's predictive power and its relevance to empirical patterns.

The agreement of our model predictions with our data on *Daphnia* and that from other studies suggests that the simple classification of dietary constraints as either affecting energy uptake or expenditure is a promising avenue for exploring the TSR within complex variations of the nutritional environment. In our model, food quality constraints are simply implemented as variability in maintenance costs (Ruiz et al. 2021) without accounting for temperature‐specific nutritional requirements (Koussoroplis et al. 2022; Masclaux et al. 2009; Ruiz et al. 2020). Furthermore, animals can respond to energy limitation by depressing their maintenance metabolism (Auer et al. 2015; Auer et al. 2016; Burton et al. 2011; Norin and Metcalfe 2019). To better predict size‐age patterns, future studies should investigate how diet composition and energetic restriction affect the thermal sensitivity of maintenance metabolic rate.

### 4.2 TSR in a Thermally Variable Environment

Thermal variability, like other environmental factors, plays a pivotal role in shaping the energy budget of ectotherms by influencing both energy intake and expenditure (Kingsolver and Woods 2016; Stocker et al. 2024; Vasseur et al. 2014). However, despite extensive research on the TSR, the specific effects of thermal fluctuations on TSR expression remain largely unexplored. Our model predicts that thermal variance shifts the temperature window in which the TSR emerges towards colder temperatures. This shift can be interpreted through the lens of Jensen's inequality (Jensen 1906), which explains how nonlinear relationships between performance and temperature interact with variance. At colder temperatures, organisms operate within the convex region of the thermal performance curve (TPC), where fluctuations tend to increase net energy gain. This enhanced energy budget promotes earlier maturation at larger sizes relative to constant conditions, thus extending TSR patterns to lower temperatures. In contrast, near the thermal optimum (*ca*. 24°C for the studied *Daphnia* clone; Van Baelen et al. 2024), performance lies in the concave portion of the TPC. Here, thermal variability reduces net energy gain, leading to smaller size‐at‐maturity and altering the temperature threshold at which growth rate declines. As a result, the TSR may vanish at temperatures where it would otherwise be expressed under stable conditions. Overall, thermal variance compresses and shifts the TSR window, reducing the range of conditions under which this pattern can be observed.

### 4.3 Size‐At‐Age Patterns in a Co‐Varying Environment

While the TSR has traditionally been studied in response to temperature alone, our findings highlight the importance of accounting for other environmental factors that covary with temperature, particularly those having a strong influence on the energy budget. Even relatively modest changes in the nutritional environment (e.g., 3% decrease in food quantity or quality per °C) are sufficient, when combined with warming, to significantly alter size‐at‐age trajectories. At colder temperatures, this covariance can shift the response from a reverse‐TSR to a conventional TSR—or vice versa. At warmer temperatures, it may weaken or even disrupt the TSR, resulting in non‐TSR patterns characterised by decreasing size‐at‐maturity and increasing age‐at‐maturity. This shift likely occurs because organisms are pushed towards their energetic and physiological limits. Once minimum size thresholds for maturation are reached, further reductions in size are no longer viable. The only remaining option is to delay maturation—resulting in older and smaller individuals (Nilsson‐Örtman and Rowe 2021). Although the interaction between thermal variance and mean temperature has a subtler impact than nutritional context alone, it still modulates size‐age responses. When all environmental cofactors vary simultaneously, the effects compound: changes in size‐ and age‐at‐maturity become more pronounced, and under particularly stressful conditions, individuals may fail to reach maturity at all.

These findings underscore the necessity of considering covariance among environmental drivers when predicting size‐at‐age responses to climate change. Both thermal variance and food availability and quality are expected to shift alongside mean temperatures (Bernhardt et al. 2020; Birnie‐Gauvin et al. 2017; Dillon et al. 2016; Hallam and Harris 2023; Hardison and Eliason 2024; Vasseur et al. 2014) meaning that canonical patterns like the TSR may no longer hold under realistic future scenarios.

## 5 Conclusion

Mechanistic models, such as those based on DEB theory, offer valuable tools for predicting organismal responses to multifactorial environmental changes. By integrating the simultaneous effects of mean temperature, thermal variability, and nutritional constraints, our study provides new insights into the future of size‐ and age‐at‐maturity dynamics under climate warming. Our results indicate that the TSR might not be robust to realistic environmental complexity. As conditions become more energetically restrictive, growth slows, and organisms tend to mature not only smaller, but also later. These predictions are supported by empirical observations in nature (Bigler et al. 1996; Doan et al. 2019; Morita and Fukuwaka 2007; Otero et al. 2012) and may represent a common response to a warmer, more variable, and resource‐limited world.

## Author Contributions

All authors have designed the work. Nathan Frizot contributed to the experimental data acquisition. Apostolos‐Manuel Koussoroplis and Nathan Frizot contributed to the conception of the model. All authors contributed to data set analysis and interpretation of data. Nathan Frizot wrote the first draft of the manuscript, and all authors contributed substantially to subsequent versions. Alexandre Bec and Apostolos‐Manuel Koussoroplis supervised the project. AI was used under author supervision for text editing.

## Funding

This work was supported by Agence Nationale de la Recherche, ASAP (ANR‐22‐CE02‐0005).

## Conflicts of Interest

The authors declare no conflicts of interest.

## Supporting information


**Data S1:** ele70273‐sup‐0001‐Supinfo.docx.

## Data Availability

The data that support the findings of this study are openly available in Dryad at https://doi.org/10.5061/dryad.xgxd254sf.
