# Supplementary material for: Ectotherm Size‐ and Age‐At‐Maturity in a Warmer, Variable and Resource‐Poor World
Source: Ecol Lett. 2025 Dec 2;28(12):e70273. doi: 10.1111/ele.70273 (PMC12670362; doi:10.1111/ele.70273)
Supplement: Supplementary file 1 — Data S1: ele70273‐sup‐0001‐Supinfo.docx. [file ELE-28-0-s001.docx]

**SUPPLEMENTARY INFORMATION**

**Extended Table 1: Predicted and observed percentage difference of mass-, age-at-maturity and growth rate between 20° and 28°C.** The reference represents treatment with high food quality (*Cryptomonas sp.*) saturating food concentration (3 mgC.l^-1^) and constant mean temperatures. We first tested scenarios were only the temperature increases, then we tested scenarios where environmental factors evolve along temperature, with the reference at 20°C to all different combinations of evolving factors at 28°C. The confidence interval of 95% has been estimated by bootstrap method for experimental data.

|  |  | | **Mass-at-maturity change (%)** | | **Age-at-maturity change (%)** | | **Growth rate change (%)** | |
| --- | --- | --- | --- | --- | --- | --- | --- | --- |
|  | 20° 🡪 28° | | Model | Experiment | Model | Experiment | Model | Experiment |
| **Only temperature increases** | Reference | | -35.6 | [-17 ; -9.6] | -29 | [-38.4 ; -38.4] | +20.7 | [+51.3 ; +55.4] |
|  | Thermal variance context | | -39.7 | [-40.2 ; -31.9] | -16.8 | [-8.6 ; -3] | +1.1 | [-13.9 ; -7.5] |
|  | Low quality context | | -37 | [-11.2 ; +6.4] | -9.3 | [-33.8 ; -26.2] | -7.4 | [+34.3 ; +48.8] |
|  | Low concentration context | | -29.6 | [-17.4 ; +2.4] | -8.3 | [-24 ; -4.3] | -4.8 | [-0.2 ; +20.6] |
| **Temperature increases associated with** | Reference | 🡪 higher variance | -43.9 | [-51.1 ; -44.8] | -16.7 | [-17 ; -12.1] | -1.2 | [-10.8 ; -4.5] |
|  |  | 🡪 lower quality | -49.7 | [-45.1 ; -36.9] | -1.9 | [+7.4 ; +11.7] | -19.5 | [-26.9 ; -22.2] |
|  |  | 🡪 lower concentration | -58.2 | [-62 ; -56.2] | +46.7 | [-3.9 ; +9.8] | -50.1 | [-42.5 ; -35.6] |
|  |  | 🡪higher variance and lower quality | -59.4 | [-52.3 ; -43.3] | +44.7 | [+71.2 ; +80.9] | -50 | [-56.9 ; -53.6] |
|  |  | 🡪 higher variance and lower concentration | -63 | [-63.2 ; -55.2] | +104.2 | [+7 ; +25.9] | -65.9 | [-49.4 ; -39.8] |
|  |  | 🡪 lower quality and concentration | / | [/] | / | [/] | / | [/] |
|  |  | 🡪 higher variance, lower quality and concentration | / | [/] | / | [/] | / | [/] |

**Extended Table 2: Type III Anova table results and parameter estimates for the model for mass-at-maturity analysis**

|  | **Df** | **SS** | **MS** | **F value** | **p-value** | |  |
| --- | --- | --- | --- | --- | --- | --- | --- |
| **Temperature** | 1 | 8724 | 8724 | 11.67 | 0.0007 | | *** |
| **Quality** | 1 | 199305 | 199305 | 266.50 | 0.0001 | | *** |
| **Quantity** | 1 | 11051 | 11051 | 14.78 | < .0001 | | *** |
| **Variance** | 1 | 112959 | 112959 | 151.04 | < .0001 | | *** |
| **Temperature:Quality** | 1 | 35782 | 35782 | 47.85 | < .0001 | | *** |
| **Temperature:Quantity** | 1 | 14094 | 14094 | 18.85 | < .0001 | | *** |
| **Temperature:Variance** | 1 | 9652 | 9652 | 12.91 | 0.0004 | | *** |
|  | **Estimate** | | **SE** | **t** | **p-value** | | |
| **(Intercept)** | 84.76 | | 15.40 | 5.50 | 0 .0008 | *** | |
| **Temperature28** | 39.42 | | 9.96 | 3.96 | < .0001 | | *** |
| **QualityCrypto** | 85.99 | | 4.93 | 17.43 | < .0001 | | *** |
| **Quantity3** | 42.57 | | 5.23 | 8.14 | < .0001 | | *** |
| **VarianceVar** | -26.17 | | 3.77 | -6.94 | < .0001 | | *** |
| **Temperature28:QualityCrypto** | -50.97 | | 7.37 | -6.92 | < .0001 | | *** |
| **Temperature28:Quantity3** | -34.96 | | 8.05 | -4.34 | < .0001 | | *** |
| **Temperature28:VarianceVar** | -21.79 | | 6.07 | -3.59 | 0.0004 | | *** |

**Extended Table 3: Type III Anova table results and parameter estimates for the model for age-at-maturity analysis**

|  | | **df** | **F value** | **p-value** |  |
| --- | --- | --- | --- | --- | --- |
| **(Intercept)** | | 1 | 47.54 | < .0001 | *** |
| **Temperature** | | 1 | 1123.19 | < .0001 | *** |
| **Quality** | | 1 | 1098.26 | < .0001 | *** |
| **Quantity** | | 1 | 120.65 | < .0001 | *** |
| **Variance** | | 1 | 135.95 | < .0001 | *** |
| **Temperature:Quality** | | 1 | 6.77 | 0.0097 | ** |
| **Temperature:Quantity** | | 1 | 4.29 | 0.039 | * |
| **Temperature:Variance** | | 1 | 679.35 | < .0001 | *** |
|  | **Estimate** | **SE** | **t** | **p-value** |  |
| **(Intercept)** | 14.93 | 1.52 | 9.85 | < .0001 | *** |
| **Temperature** | -0.07 | 0.06 | -1.19 | 0.23 |  |
| **QualityCrypto** | -1.97 | 0.75 | -2.61 | 0.0094 | ** |
| **Quantity** | -1.25 | 0.37 | -3.34 | 0.0009 | *** |
| **VarianceVar** | -5.45 | 0.23 | -23.78 | < .0001 | *** |
| **Temperature:QualityCrypto** | -0.08 | 0.03 | -2.38 | 0.0177 | * |
| **Temperature:Quantity** | -0.04 | 0.01 | -2.87 | 0.0043 | ** |
| **Temperature:VarianceVar** | 0.24 | 0.01 | 26.06 | < .0001 | *** |

**Extended Table 4: Type III Anova table results and parameter estimates for the model for growth rate analysis**

|  | | **df** | **F value** | **p-value** |  |
| --- | --- | --- | --- | --- | --- |
| **(Intercept)** | | 1 | 433.94 | < .0001 | *** |
| **Temperature** | | 1 | 616.46 | < .0001 | *** |
| **Quality** | | 1 | 5769.27 | < .0001 | *** |
| **Quantity** | | 1 | 545.76 | < .0001 | *** |
| **Variance** | | 1 | 37.45 | < .0001 | *** |
| **Temperature:Quality** | | 1 | 39.19 | < .0001 | *** |
| **Temperature:Quantity** | | 1 | 33.60 | < .0001 | *** |
| **Temperature:Variance** | | 1 | 573.41 | < .0001 | *** |
|  | **Estimate** | **SE** | **t** | **p-value** |  |
| **(Intercept)** | 0.195 | 0.074 | 2.63 | 0.0089 | ** |
| **Temperature** | -0.005 | 0.003 | -1.61 | 0.1072 |  |
| **QualityCrypto** | 0.062 | 0.033 | 1.87 | 0.0615 | . |
| **Quantity** | -0.123 | 0.022 | -5.53 | < .0001 | *** |
| **VarianceVar** | 0.700 | 0.030 | 22.94 | < .0001 | *** |
| **Temperature:QualityCrypto** | 0.010 | 0.001 | 6.79 | < .0001 | *** |
| **Temperature:Quantity** | 0.009 | 0.001 | 8.23 | < .0001 | *** |
| **Temperature:VarianceVar** | -0.034 | 0.001 | -23.95 | < .0001 | *** |


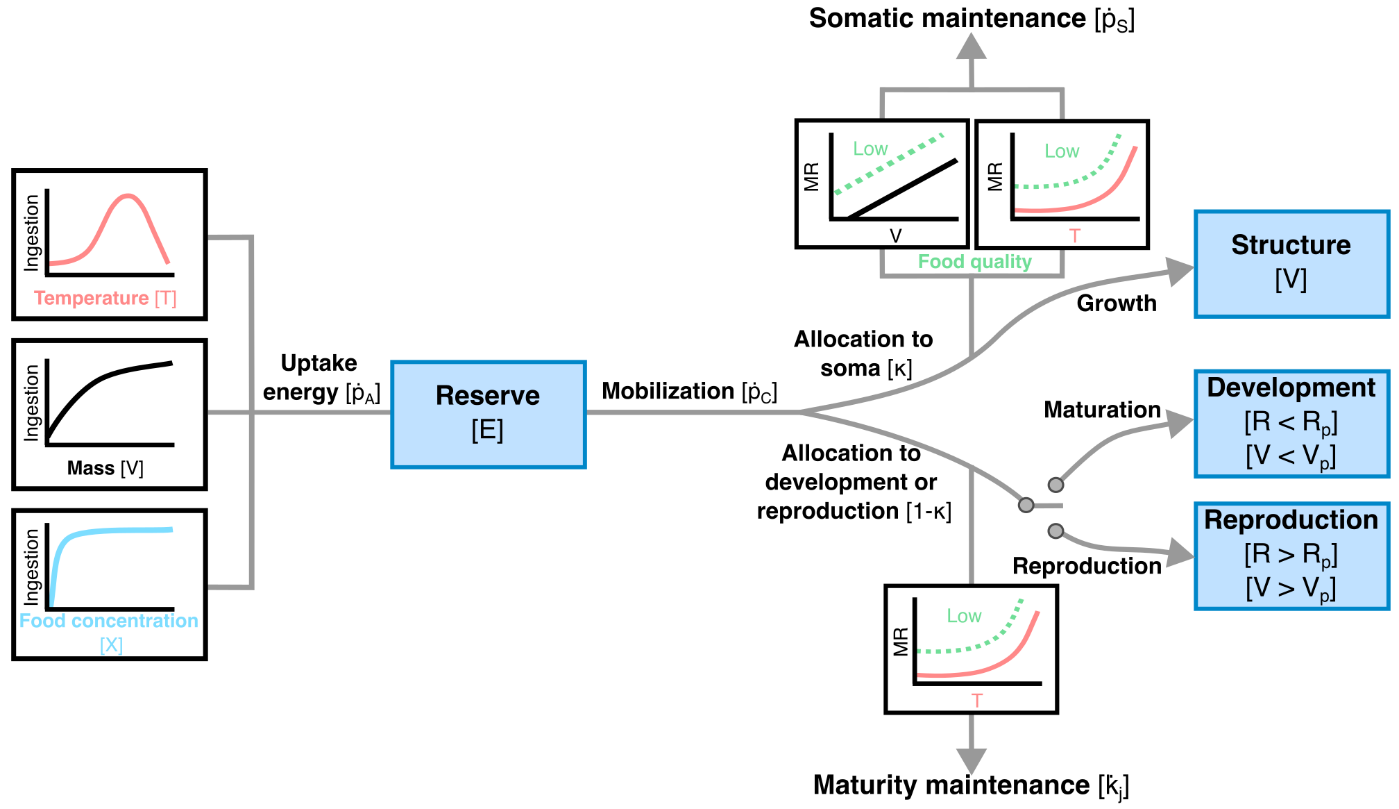


**Extended Fig. 1: Diagram of main components of the model**. Blue boxes are the state variables of the model. White boxes indicate the dependance of energy fluxes on state or forcing variables. Red lines represent functions dependent on temperature, blue line represents function dependent on food concentration, and green dashed lines represent how functions are influenced by low a food quality. See **Methods** and **Table 1** for detailed model description and equations**.**

**
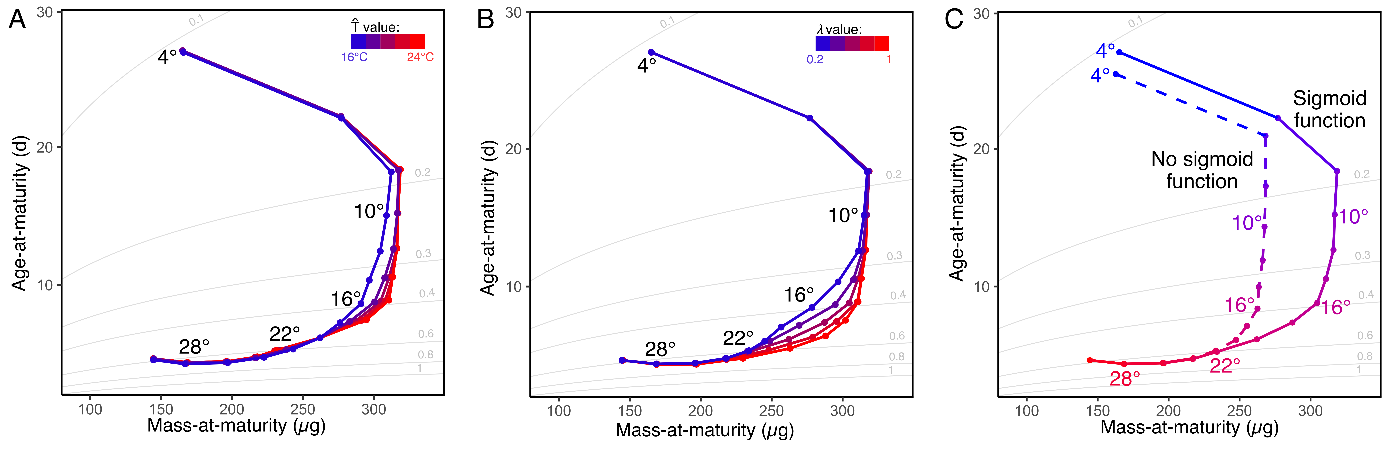
**

**Extended Fig. 2:** **Sensitivity analysis of** $\hat{\boldsymbol{T}}$**,** $\boldsymbol{\lambda}$ **and of the sigmoid function of the model.** **A.** The temperature of inflexion $\hat{T}$is tested from 16° (blue) to 24°C (red), the arbitrarily chosen value used in the model was 20°C. **B.** The parameter controlling the stiffness of the transition of the equation $\lambda$ is tested from 0.2 (blue) to 1 (red), the arbitrarily chosen value used in the model was 0.6. **C.** In the model, equation 10 uses a sigmoid function, here we tested the effect of a simple linear function instead of the sigmoid one. Isoclines represent the growth rate for all possible combinations of size and age calculated as ${(ln({mass}_{mat})-{ln(mass}_{initial}))}/{{age}_{mat}}$. Error bars indicate 95% confidence intervals.

**
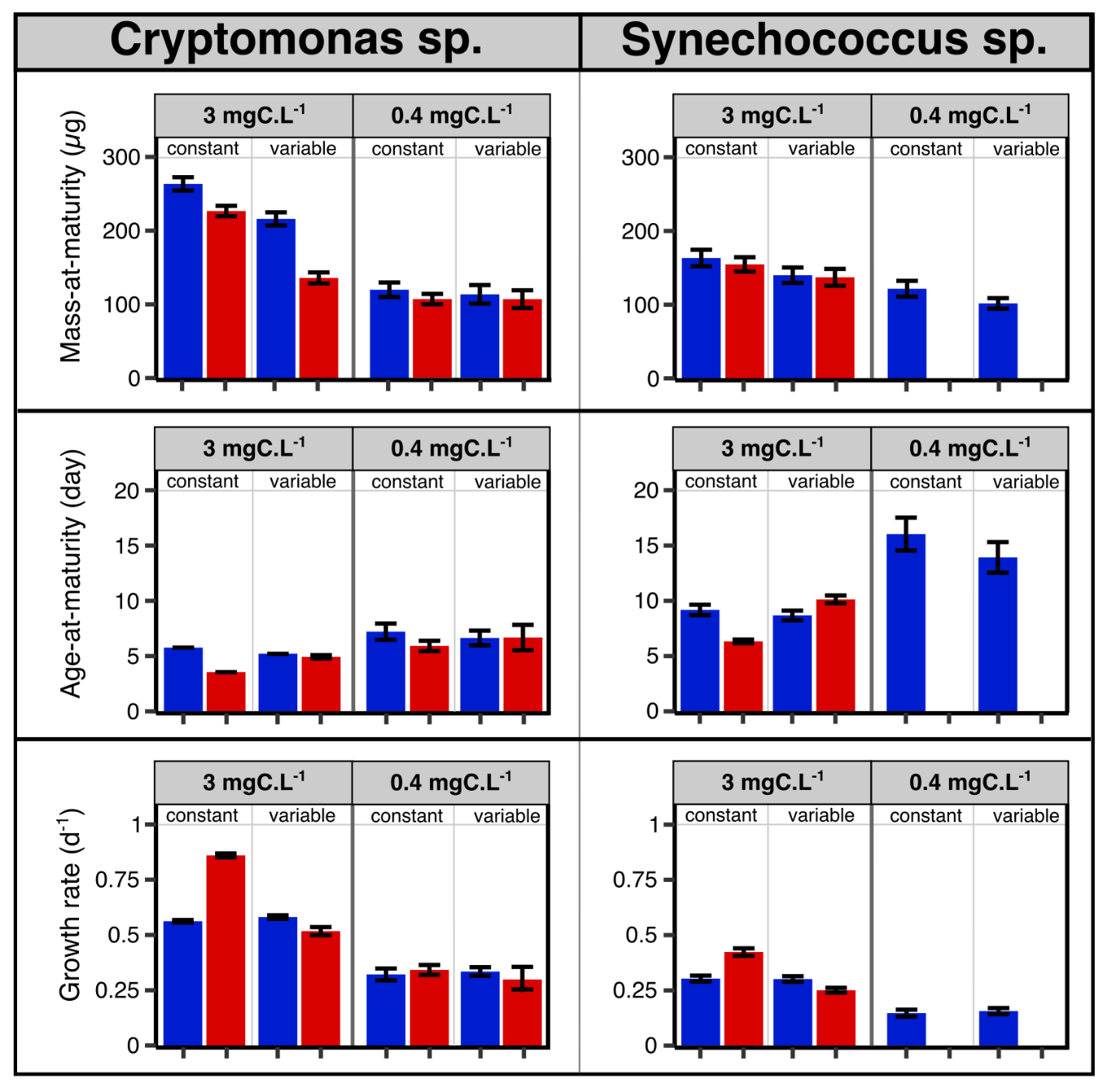
**

**Extended Fig. 3:** **Observed values of mass-, age-at-maturity, growth rate and clutch size of *Daphnia magna* to all 16 factorial combinations of tested factors.** Blue bars: 20°C; red bars: 28°C. The constant treatment represents a thermal variance with 0°C amplitude and the variable one represents an amplitude of 5°C around the mean. *Cryptomonas sp.* represents the high food quality and *Synechococcus sp.* represents the low food quality. Error bars indicate 95% confidence intervals.


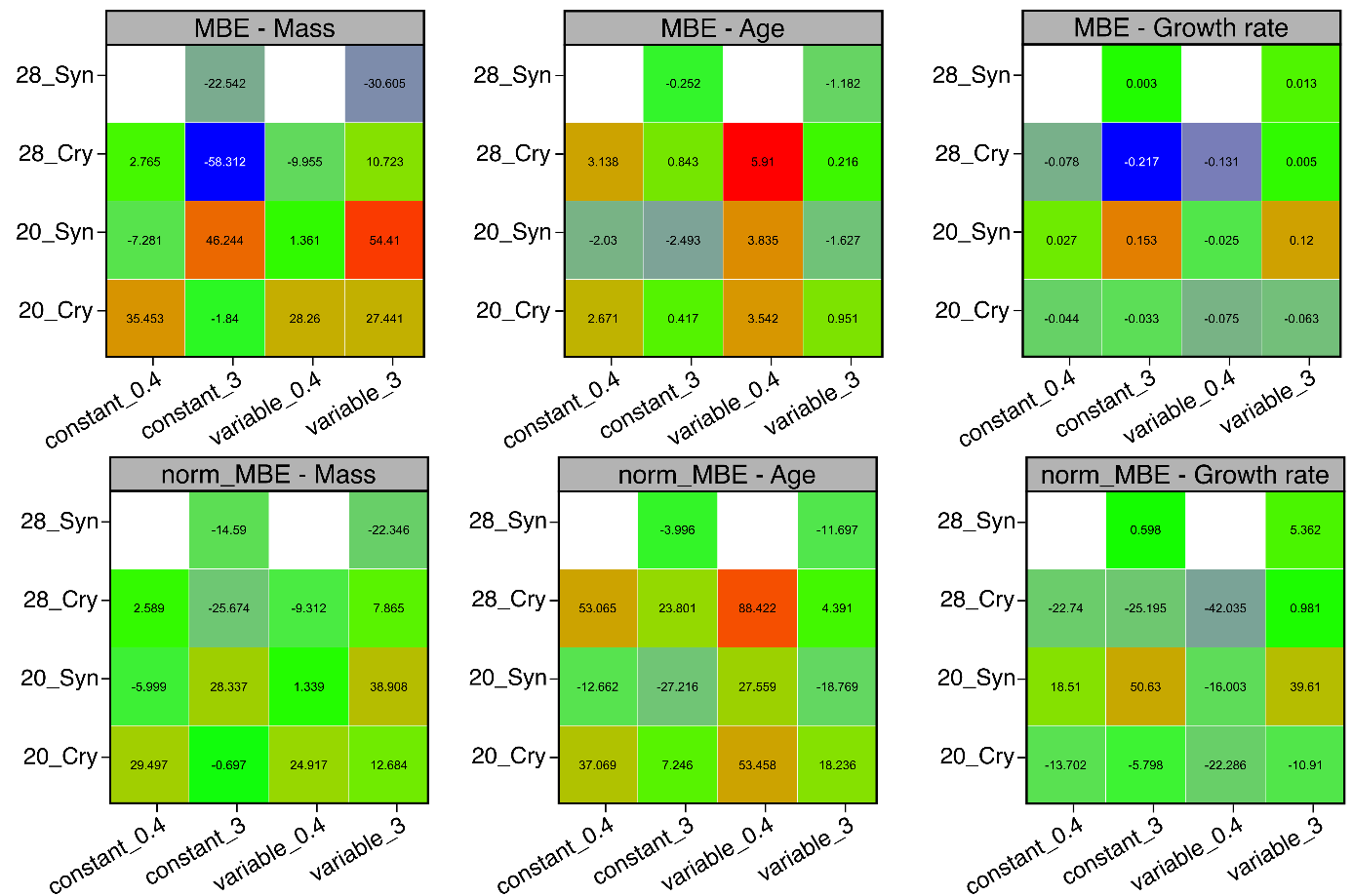


**Extended Fig. 4:** **Heatmaps of Mean Bias Error (MBE, trait-specific unit) and normalized MBE (%) between observed and predicted values of mass-at-maturity, age-at-maturity and growth rate.** Results closer to zero indicate better agreement between predicted and observed values (green). Positive results reflect overestimation of the predicted values (red), negative results reflect underestimation (blue). Axis represent the different tested treatments, with temperature (20 or 28°C), food quality (Cry = *Cryptomonas sp.*, high quality; Syn = *Synechococcus sp.*, low quality), thermal variance (constant temperature; variable temperature) and food quantity (0.4 or 3 mgC.l^-1^). The normalized MBE has been normalized by the mean of observed values.

**Extended methods: Mapping response patterns in Figure 2**

*DEB simulations*

To classify thermal response patterns (TSR, reverse-TSR, and non-TSR) across environmental contexts we first simulated size- and age-at-maturity using the Dynamic Energy Budget (DEB) model (*see* main text) across a factorial grid of ($i\times j$) mean temperature and factor of interest combinations.

Simulation grid:

-Temperature (4-30°C, by=0.1°C), $i=$ 53

-Food quantity (0.1-3.5 mgC.L^-1^, by=0.1 mgC.L^-1^): $j=$ 35

-Food quality (${[\dot{p}}_{M}]$: 315.86-754.14 J.cm^-3^.d^-1^, by=14.14 / $\dot{k}_{j}$: 0.0483-0.1017 d^-1^, by=0.0017) $j=$ 32

-Temperature amplitude (0-6°C, by=0.2°C): $j=$31

For each temperature–food combination, simulations returned either a valid outcome (size- and age-at-maturity successfully attained) or a non-maturity case (where individuals failed to reach maturity within the simulation timeframe, coded as *NA*). From these outcomes, juvenile growth rate was calculated using equation (13) in the main text, which links simulated size- and age-at-maturity to growth performance. This provided three derived traits for each grid cell: size-at-maturity, age-at-maturity, and juvenile growth rate.

*GAM fits*

To model the dependence of these traits on environmental gradients, we used generalized additive models (GAMs) implemented in the **mgcv** package in R (Wood 2017; version 1.8-42). Each response variable (size, age, growth rate) was fitted as a smooth cubic spline of temperature and the factor of interest and their tensor product interaction smooth. A maximum basis dimension ranging between 5-8 was used and models were fitted with restricted maximum likelihood (REML) estimation. The quality of model fits is presented in Extended Fig. 5.

*Estimation of local slopes and classification of response patterns.*

Local slopes of the fitted GAM surfaces with respect to temperature were estimated by the finite difference method. This method calculates numerical approximations of their first order partial derivative. Specifically, for each grid point of temperature and food quantity (or quality or temperature amplitude) $(T,F)$, the slope was computed as:

$$\frac{\partial y}{\partial T}|_{(T,F)}\approx\frac{\hat{y}\left( T+\Delta T \right)-\hat{y}\left( T-\Delta T \right)}{2\Delta T}$$

where $\hat{y}$denotes the GAM-predicted value of the response variable (size-at-maturity, age-at-maturity, or growth rate), and $\Delta T$ is a small step size in temperature (here set to 0.26°C which corresponds to 1/100^th^ of the temperature range).

Finite differences were computed across all points of the temperature–food grid separately for each trait. The resulting slope matrices (age slope, size slope, growth slope) were then used to classify thermal response patterns according to the sign combinations defined in the main text:

**TSR**: negative slope for age-at-maturity and size-at-maturity, but positive slope for growth rate.

**Reverse-TSR**: negative slope for age-at-maturity, positive slope for size-at-maturity and growth rate.

**Non-TSR**: any other combination of slope signs.


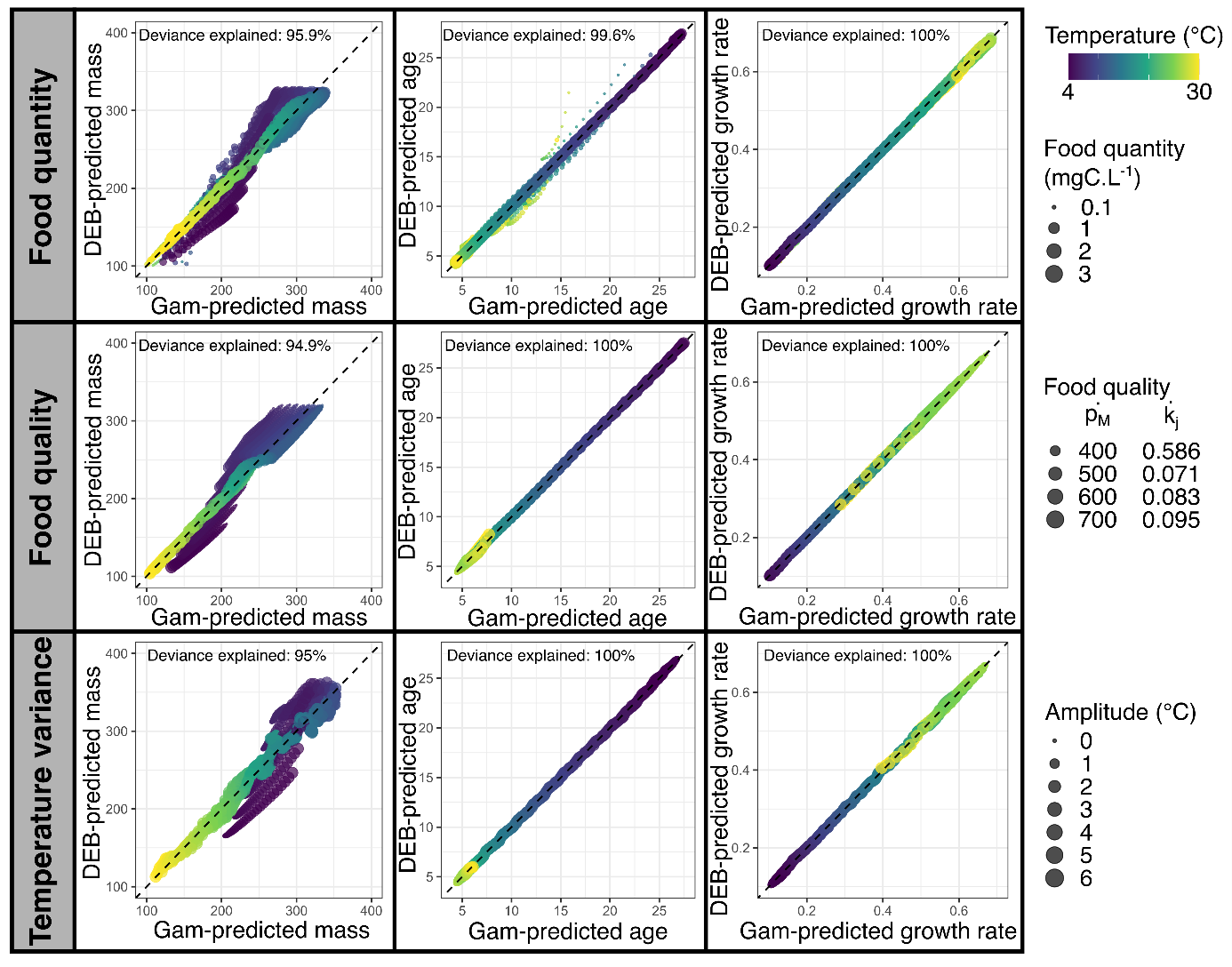


**Extended Figure:** **DEB model-predicted results vs GAM-predicted results.** DEB-predicted data is represented on the y-axis and GAM-predicted data is represented on the x-axis. The black dashed line represents the 1:1 ratio; points closer to this line indicate a stronger agreement between DEB and GAM predictions. The color gradient represents temperature and the gradient in dot size represents the value of the varying parameter.
